# DEVELOPMENT AND APPLICATION OF R1RHO DISPERSION IMAGING OF AGING MUSCLE IN AN ANIMAL MODEL

**DOI:** 10.1093/geroni/igad104.3270

**Published:** 2023-12-21

**Authors:** Fatemeh Adelnia, Feng Wang, John Gore

**Affiliations:** Vanderbilt University Medical Center, Nashville, Tennessee, United States; Vanderbilt University Medical Center, Nashville, Tennessee, United States; Vanderbilt University Medical Center, Nashville, Tennessee, United States

## Abstract

Compelling evidence from animal studies demonstrates that microvascular impairment contributes to the etiologies of several age-related chronic diseases, including sarcopenia. For example, capillary hemodynamic profiles are altered in old F344 rat spinotrapezius muscle compared with those of younger counterparts. However, a validated non-invasive measure of microvasculature geometry and structure has not yet been developed. Recently, it has been shown that R1ρ dispersion imaging at weak locking field frequencies (FSL) provides a unique way of characterizing microvessel size and density. Moreover, previous R1ρ images were acquired at strong FSL (≥500Hz) and used to measure the age-related differences linked to macromolecular deposition, such as collagen in the extracellular matrix. Here, we aimed to develop a non-invasive 3D volume R1ρ dispersion imaging method at weak FSLs (< 300Hz), to quantify microvasculature changes in skeletal muscle. We successfully implemented the protocol and applied it to the lower leg of five F334 female rats at age 15 mo. The whole lower leg of each rat was also imaged using conventional multislice T1-FLASH, Diffusion-weighted, and T2-MGE pulse sequences. We found that R1p value measured in calf muscles decreased from 46.92Hz to 41.63Hz by increasing FSL from 5Hz to 240Hz, which we suggest derives from the effects of rephasing some of the signal losses that arise from diffusion in intrinsic magnetic field gradients generated from microvessels. Future direction of this ongoing study will be to compare these results with data collected from young female rats at age 6 months and further validation using ex-vivo histology.

